# Viral-like TLR3 induction of cytokine networks and α-synuclein are reduced by complement C3 blockade in mouse brain

**DOI:** 10.1038/s41598-023-41240-z

**Published:** 2023-09-13

**Authors:** Ria Thomas, Kyle J. Connolly, Oeystein R. Brekk, Anthony J. Hinrich, Michelle L. Hastings, Ole Isacson, Penelope J. Hallett

**Affiliations:** 1 Neuroregeneration Institute, McLean Hospital/Harvard Medical School, Belmont, MA 02478 USA; 2 Center for Genetic Diseases, Chicago Medical School, Rosalind Franklin University of Medicine and Science, North Chicago, IL 60064 USA

**Keywords:** Cellular neuroscience, Neuroimmunology, Neurodegeneration, Complement cascade, RNA metabolism, Viral infection

## Abstract

Inflammatory processes and mechanisms are of central importance in neurodegenerative diseases. In the brain, α-synucleinopathies such as Parkinson’s disease (PD) and Lewy body dementia (LBD) show immune cytokine network activation and increased toll like receptor 3 (TLR3) levels for viral double-stranded RNA (dsRNA). Brain inflammatory reactions caused by TLR3 activation are also relevant to understand pathogenic cascades by viral SARS-CoV-2 infection causing post- COVID-19 brain-related syndromes. In the current study, following regional brain TLR3 activation induced by dsRNA in mice, an acute complement C3 response was seen at 2 days. A C3 splice-switching antisense oligonucleotide (ASO) that promotes the splicing of a non-productive C3 mRNA, prevented downstream cytokines, such as IL-6, and α-synuclein changes. This report is the first demonstration that α-synuclein increases occur downstream of complement C3 activation. Relevant to brain dysfunction, post-COVID-19 syndromes and pathological changes leading to PD and LBD, viral dsRNA TLR3 activation in the presence of C3 complement blockade further revealed significant interactions between complement systems, inflammatory cytokine networks and α-synuclein changes.

## Introduction

### Viral infections and consequent brain inflammation increase neurodegenerative disease risk

Inflammatory processes and mechanisms have emerged to be of central importance in the genesis of neurodegenerative diseases^1–3^. Neuroinflammation, characterized by reactive microglia and astrocytes, and elevated levels of inflammatory mediators in the brain, was traditionally viewed as secondary to neuronal death and dysfunction in Alzheimer’s disease (AD) and Parkinson’s disease (PD). Contrary to this conventional view, there is now robust evidence from preclinical and clinical studies that immune activation can contribute to and drives disease pathogenesis. Drivers of such inflammation can be cell-autonomous or the result of network interaction between neurons, glia, vascular and blood-derived agents. We and others have previously shown that bacterial, viral, lipid, or metabolic activators are potent initiators of such neuroinflammation^1,4–9^. Of particular interest are the dsRNA toll like receptor 3 (TLR3) receptor activators from viral sources that can initiate such inflammatory cascades^1, 2, 5^.

Viral and other inflammatory conditions are linked to increased risk of neurodegenerative diseases and cognitive dysfunction^1,2, 10,11^. Systemic cytokine elevations following viral infection, such as those that cause cytokine storm in patients with COVID-19, are associated with cytokine and glial activations in the brain^12–15^. Infection with Epstein-Barr virus (EBV) is associated with a 32-fold increased risk for developing multiple sclerosis, with onset of disease symptoms beginning approximately 10 years after infection^16^. Furthermore, recent epidemiological studies provide compelling evidence that repeated viral infections causing influenza drastically increase the probability (more than tenfold) of developing neurodegenerative diseases^11^. Viral outbreaks, including the 1918 influenza pandemic and periodic mosquito-borne flavivirus epidemics such as Japanese encephalitis virus and West Nile virus, have led to subsequent diagnoses of post-encephalitic parkinsonism among survivors of the incident viral infection^2,17–20^. Given the risk of neurological “long-COVID” symptoms following SARS-CoV-2 infection^21,22^, evidence of COVID-induced changes in brain regions that are related to cognition and neurological disorders^23^, and association of COVID-19 with onset of neurodegenerative disease^24^, it is highly significant to establish the underlying biological cellular brain responses that are associated with TLR3 activation and viral inflammation. In the context of historical viral outbreaks and the current COVID-19 pandemic, understanding how the long-term consequences of such viral infections could precipitate degenerative changes within vulnerable brain regions is of current and critical importance.

Several inflammatory processes have been shown to be linked to the classic pathological hallmarks of neurodegenerative diseases at post-mortem. For example, α-synuclein, which is associated with neuropathology of PD and Lewy body dementia (LBD), is increased and aggregates in response to cellular stressors, such as mitochondrial dysfunction, lipid accumulation, and inflammation^6,25–28^. Injection of the toll like receptor 4 (TLR4) agonist, lipopolysaccharide (LPS), into the midbrain of rodents induces activation of inflammatory cytokine networks^4,29^, accumulation of insoluble and aggregated α-synuclein^30^ and dopamine neuron degeneration^31^, and long-term systemic administration of low-dose LPS administration in mice induces significant reductions of dopaminergic neurons^32^. It has also been shown that α-synuclein is upregulated by certain viral infections^33–36^. Deficiency of α-synuclein in mice increases susceptibility to infection by viral or bacterial causes, suggesting that α-synuclein can participate in anti-microbial functions^37^. Importantly, α-synuclein accumulation has been reported in the brain of rodents and non-human primates following SARS-CoV-2 systemic infection^38,39^. These connections between acute α-synuclein functional responses to potent inflammatory stimuli, and pathological α-synuclein increases that lead to aggregation associated with chronic inflammatory cytokine and glial activations in brain regions vulnerable to degeneration, highlight that inflammation is a critical driver of pathology in neurodegenerative disease.

### Brain responses to viral-like TLR3 activation

The cellular milieu of the brain and periphery carries many receptors for immune activation, and it is important to understand how such receptor systems and cells can trigger neuroinflammation. Typically, evolutionarily conserved immune responses are linked to receptors specialized in recognizing bacterial and viral components. TLRs recognize specific pathogen and damage-associated molecular patterns. The function of TLRs is to surveil extracellular and intracellular environments for signs of infection or damage and to signal the activation of innate immune and inflammatory responses to clear infection and remove apoptotic cells. The up-regulation of TLRs has been demonstrated in brains with α-synucleinopathies, such as PD, LBD, and multiple system atrophy (MSA)^40–44^. For these reasons, it is of critical importance to experimentally model conditions that mimic viral-like activation of receptors designated for immune activation. TLR3 specifically recognizes and is activated by dsRNA, which are intermediates of viral replication, and signals downstream anti-viral immune and inflammatory responses. Brain TLR3 activation has previously been modelled in vivo by direct administration of the synthetic viral-like dsRNA mimetic, Poly(I:C), into rodent brain^5,45–48^. TLR3 activation by the administration of Poly(I:C) into substantia nigra and striatum led to a timeline of neuroinflammatory responses, characterized by activation of astrocytes and microglia within 4 days that continued to increase until peak activation at 12 days during cytokine storm, and persisted for 33 days^5,46,47^. While TLR3 activation by itself was not sufficient to induce neurodegeneration, priming with such viral mimetics exacerbated the degeneration of nigral dopaminergic neurons to subsequent injection of 6-OHDA^5^ and to a small molecule inducer of α-synuclein fibril formation^48^, showing that brain inflammation increases the vulnerability of neurons to additional stress. Neutralizing the Poly(I:C) induced brain inflammation by systemic administration of IL-1 receptor antagonist protected the nigral dopaminergic neurons from the damage caused by TLR3 activation combined with 6-OHDA^5^, demonstrating that persistent inflammation of the brain increases neuronal vulnerability to additional damage, while reducing inflammation promotes neuronal cell resilience. The clear evidence for long-term brain inflammatory and degenerative sequelae to viral infections, which has led to subsequent neurological diagnoses such as post-encephalitic parkinsonism, supports the hypothesis and findings that preventing brain inflammatory responses caused by viral-like TLR3 activation could be protective^1,5,27^.

### Targeting the complement pathway to reduce inflammatory activations

In the sequence of events that typically follow viral or bacterial perturbations, there are well-known activation cascades including the innate acute response involving complement factors. The complement system, an integral part of the innate immune system, plays a vital role in clearance of pathogens by mounting an inflammatory response. Additionally, two components of this pathway, C1q and C3, have been identified to play beneficial role in microglial regulation of synaptic pruning during neural development^49,50^ and excessive pruning in adult brain have been implicated in neurodegeneration. Complement can be activated by three different pathways, a C1q-dependent classical pathway, and C1q-independent alternative and lectin pathways. All three activation axes converge on the enzymatic cleavage of complement factor C3 into C3a and C3b fragments. C3b in conjunction with other proteins lead to cleavage of C5 into C5a and C5b. C5b along with C6 through C9, constitute the terminal lytic pathway that leads to the formation of the membrane attack complex (MAC)^51^. The three main effector functions of the complement cascade include opsonization of C3b, C4 and C1q tagged target cell, immune cell recruitment to the site of injury by the anaphylatoxins C3a and C5a, and lysis of the target cell by the formation of MAC^52^. TLRs and the complement cascade are both part of the innate immune system and respond to pathogenic infections, and interactions between them are plausible.

It is of great interest to explore the influence of complement system blockade in reducing brain inflammatory processes and factors, such as α-synuclein in PD and LBD. With the presence of multiple activation pathways and downstream effects, the complement system can be targeted at several levels depending on the desired outcome. While blocking action of the anaphylatoxins C3a by the C3aR antagonist SB290157^53^, and C5a by the C5aR antagonists PMX53 and PMX205^54^, will inhibit inflammation and opsonization of target cells, targeting C5b, and or C6 through C9 would block the terminal lytic pathway^55^. Being at the convergence point of multiple complement activating pathways, C3 is a good target to manipulate all branches of the complement cascade, and this was the approach utilized in this study.

The current study utilized a rodent model of TLR3 activation in combination with a C3 splice-switching antisense oligonucleotide (ASO) specific to complement C3, to reduce C3 expression in the brain in vivo. The aim of this study was to experimentally model innate brain immune responses following TLR3 viral-like activation, and to examine how blocking complement C3 activation during TLR3 stimulation can reduce acute cellular and inflammatory responses downstream of TLR3. Mice received two brain injections of C3 ASO or control ASO at separate timepoints, followed by injection of dsRNA (PolyI:C) to activate brain TLR3 receptors (see schematic in Fig. 2A). Changes in brain levels of complement pathway proteins, cytokines and α-synuclein were examined in the acute phase of TLR3 activation at 2 days following injection of dsRNA.

## Materials and methods

### Animals

Male and female C57BL/6NJ mice (strain #005304, Jackson Laboratories) were used for all experiments. Animals were housed in standard conditions in a 12 h dark and 12 h light cycle with ad libitum access to food and water. Experimental group sizes of n = 9–10 mice were used, except where explicitly stated. All animal procedures were performed in accordance with current National Institutes of Health and ARRIVE (Animal Research: Reporting of In Vivo Experiments) guidelines and were approved by the Institutional Animal Care and Use Committee at McLean Hospital/Harvard Medical School.

### Neonatal injection of NT and C3 ASO

Phosphorodiamidate morpholino oligomer that is complimentary to the 5’ splice site of C3 exon 2 (C3 ASO: 5’-TAGTTCCAGTTCCTTGCCCACCTTG-3’) was purchased from Gene Tools, LLC. A non-target (NT) standard control oligonucleotide (5'-CCTCTTACCTCAGTTACAATTTATA-3') was used as the negative control (Gene Tools, LLC). Neonatal mouse pups (P1-P2) received i.c.v. injection of ASO as described previously^56,57^. A 10 μl Hamilton syringe with a 33-gauge needle, point style 4, angle 30° was used for injection. Pups were briefly cryoanesthetized and received a 2.5 μl i.c.v. injection of 20 μg/μl NT or C3 ASO (50 μg dose) (Gene Tools) in sterile 0.9% saline with 0.01% Fast Green FCF in the left ventricle. The left ventricle was targeted by injecting 2.5 mm anterior to the lambda suture, 1 mm lateral to the sagittal suture and to a depth of 2 mm.

### Stereotaxic injection of ASO and Poly(I:C) in the striatum of adult mouse brain

Stereotaxic coordinates for injection into the mouse striatum were described by our lab previously^58^. Anesthesia was induced before surgery using 5% isoflurane at 1 ml/min for 3 min, and then maintained throughout surgery using 1.5% isoflurane at 1 ml/min. Mice were secured in a stereotaxic frame (Stoelting) and injected using a 10 μl Hamilton syringe with a 31-gauge needle. For the delivery of ASO, mice aged 8–9 weeks received three 1 μl deposits of 40 μg/μl NT or C3 ASO (40 μg/site) (Gene Tools) in sterile 0.9% saline into the right striatum at a rate of 0.5 μl/min using a microinfusion pump (Stoelting), with a 4 min wait time before needle retraction. Three ASO deposits were delivered at the following anteroposterior (AP), mediolateral (ML) and dorsoventral (DV) coordinates: (1) AP, + 1.4 mm; ML,  − 1.5 mm; DV,  − 2.5 mm; (2) AP, + 0.1 mm; ML,  − 2.0 mm; DV,  − 3.5 mm; (3) AP, + 0.1 mm; ML, − 2.0 mm; DV, − 2.2 mm. Two weeks after receiving ASO, mice received a single 3 μl deposit of 16.7 μg/μl Poly(I:C) (50 μg/site) (Axxora) in ddH_2_O, or vehicle (3 μl ddH_2_O), delivered into the right striatum at a rate of 0.5 μl/min, with a 4 min wait time before needle retraction. Vehicle and Poly(I:C) solutions were warmed to 37 °C before injection. Poly(I:C) was delivered using the following coordinates: AP, + 1.4 mm; ML,  − 1.5 mm; DV,  − 2.7 mm. AP and ML coordinates were relative to bregma, DV coordinates were relative to dura. Mice injected with Poly(I:C) or vehicle were perfused with cold phosphate-buffered saline (PBS) 2 days post injection. The brain was isolated, and the right (injected) striatum was immediately dissected out, snap-frozen, and stored for subsequent transcript and protein analysis.

### RNA isolation and C3 expression analysis by RT-PCR

Striatum tissues were homogenized in RIPA buffer (Thermo Scientific) supplemented with Halt protease and phosphatase inhibitor cocktail (Thermo Scientific, #1861284) and EDTA (Thermo Scientific, #1861283). 1 ml of TRIzol reagent (Thermo Scientific, #15596026) was added to 50ul of the lysate and RNA was isolated by standard phenol:chloroform phase separation and ethanol precipitation (Qiagen). RNA was reverse transcribed using a GoScript reverse transcription system (Promega). Radiolabeled PCR was carried out as previously described^56^ using mouse-specific *C3* primers (mC3ex1F, 5′-AGCTACTAGTGCTACTGC-3′ and mC3ex3R, 5′-GGTACCCACTCTGGAAGC-3′) and *bActin* primers: mB-actinEx2F: 5’-GGCTGTATTCCCCTCCATCG-3’ and mB-actinEx3R: 5’-CCTGTTGGTAACAATGCCATGT-3’.

### Quantitative RT-PCR

cDNA was synthesized using QuantiTect Reverse Transcription kit (Qiagen, #205311) according to manufacturer’s instructions. qPCR reactions were performed using Power SYBR**®** Green PCR master mix (Thermo Fisher, #4367659) and commercial primers (all Qiagen QuantiTect Primer Assays: *C3*, #QT00109270; *Il-6*, #QT00098875; *Tnfa*, #QT00104006; *Ifng*, #QT02423428; *Ccl5*, # QT01747165; *Ccl2*, # QT00167832; *Il-1a*, #QT00113505). Five ng of input cDNA was used for each reaction and *bActin* was used as the reference gene (QuantiTect Primer Assay, #QT01136772). qPCR reaction was run on a StepOnePlus real time PCR system (Applied Biosystems) and analysis was performed using the 2^-ΔΔC(T)^ method^59^.

### Immunoblotting

For protein extraction, crude tissue homogenates in RIPA buffer were briefly sonicated and clarified by centrifugation at 15,000 × *g* for 10 min at 4 °C. Supernatant fractions were stored at  − 30 °C for subsequent protein analysis. Protein concentrations in the supernatant fractions were quantified by bicinchoninic acid (BCA) assay (Pierce). Sample protein concentrations were equalized in ddH_2_O and proteins were denatured by incubation in reducing sample buffer for 5 min at 95 °C (complement C3) or 70 °C (all other target proteins). Ten μg of protein were separated by SDS-PAGE on Criterion precast stain-free SDS 4–15% polyacrylamide gradient gels (Bio-Rad, #5678085). Total protein loading was imaged in-gel by stain-free UV-fluorescence using a ChemiDoc XRS with Image Lab software (Bio-Rad). Proteins were transferred to polyvinylidene fluoride (PVDF) membranes by semi-dry transfer at 20 V and 2.5 A for 7 min using a Trans-Blot Turbo system (Bio-Rad). Membranes were blocked for 30 min with 5% milk in Tris-buffered saline containing 0.1% Tween 20 and were probed overnight at 4 °C with primary antibodies against the following target proteins: α-synuclein (BD biosciences, BD610787 1:2000), C3 (Abcam, ab200999, 1:1000), C1q (Abcam, ab182451, 1:500), Iba1 (Abcam, ab178846, 1:1000), GFAP (EMD Millipore, MAB360, 1:2000), GAPDH (Cell Signalling Technology, #2118, 1:2000). Membranes were incubated with HRP-conjugated secondary antibodies for 1 h at room temperature. Target proteins were visualized using ECL substrate (Thermo Scientific) to generate chemiluminescent signal that was imaged using a ChemiDoc XRS system. Protein levels were quantified by densitometry analysis using Image Lab software.

### Statistical analysis

Statistical data analyses were performed in GraphPad Prism software version 9.3.1. All data are expressed as arithmetic mean $\pm$ SEM. Unpaired two-tailed student’s t-test or one-way ANOVA followed by Tukey’s post hoc testing was used as appropriate and the test used for each analysis is mentioned in the figure legend. Correlation analyses were performed by Pearson’s correlation analysis to determine correlation coefficients (r). In all cases, outliers identified using the iterative Grubb's function in GraphPad Prism, with alpha set at 0.05, were removed from subsequent analyses. *P* value < 0.05 was considered significant for all analyses.

## Results

### Striatal TLR3 activation by dsRNA delivery causes increases in complement C3 and α-synuclein protein levels

The present study addressed acute changes following brain TLR3 activation and potential interventions to reduce potential pathogenic effects in the brain. Preliminary work showed that TLR3 activation in the CNS by intra-cranial administration of Poly(I:C) (dsRNA) leads to significant local upregulation of complement C3 at 2 days post injection (Fig. S1 A-B). This response subsided by 12 days after TLR3 activation, indicating that complement pathway activation might have a role in the acute response to TLR3 activation in the CNS. To further understand the acute responses to TLR3 activation in the brain, the current study utilized intra-striatal administration of Poly(I:C) to characterize these changes after 2 days in vivo.

**Figure 1:**
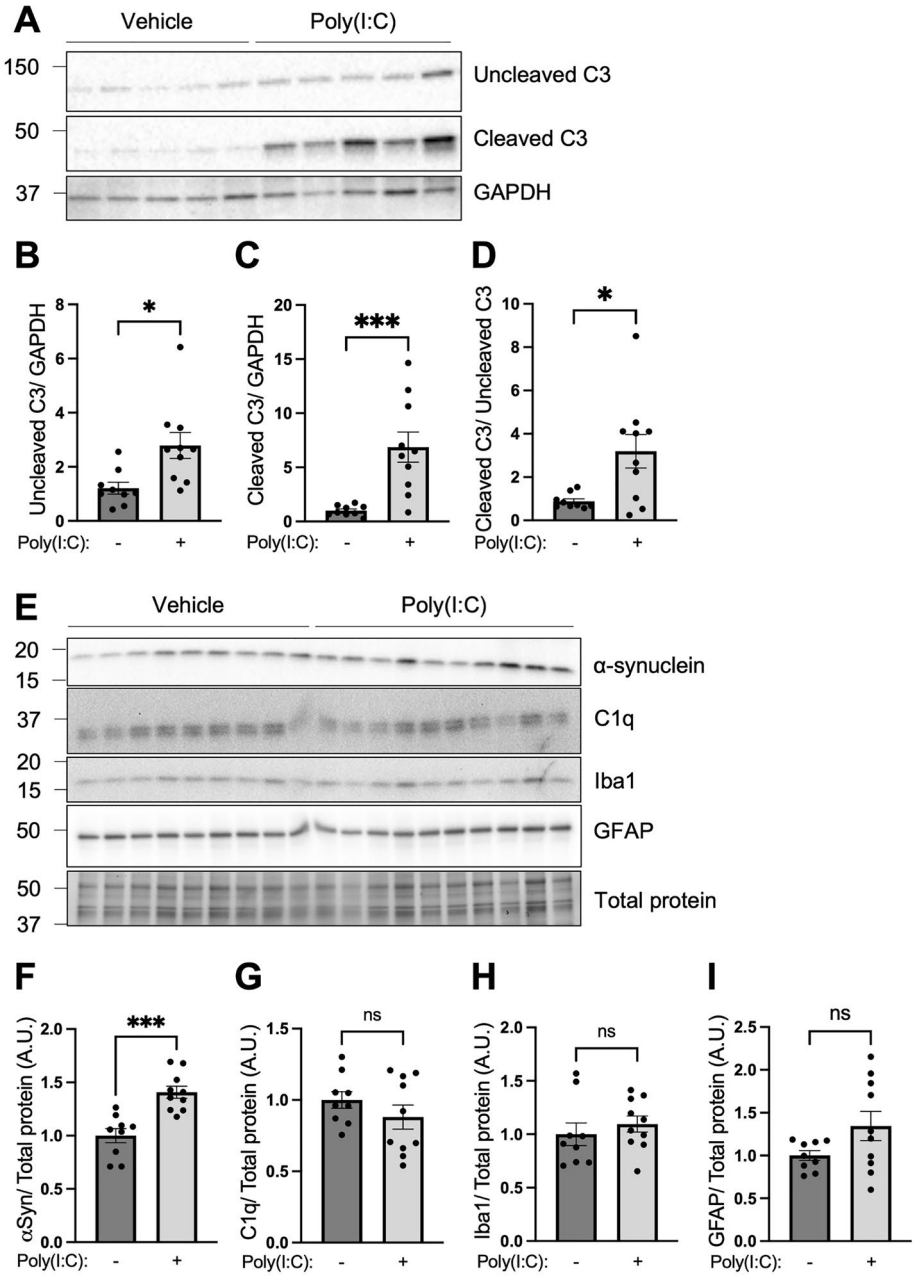
Intra-striatal TLR3 activation in mouse brain increased complement C3 and α-synuclein protein levels. WT mice received a single unilateral deposit of 50 μg Poly(I:C) (n = 10) or vehicle (n = 9) in the striatum. Two days later, injected striata were isolated and homogenised in RIPA buffer for protein detection by Western blot (**A, E**). (**B**–**D, F**–**I**) Histogram bars represent the mean levels of target protein normalized to GAPDH or total protein visualized by stain-free UV-fluorescence. Uncropped blots can be found in Figs. S3–S7. Error bars represent SEM. A.U., arbitrary units. Statistical analysis was performed using unpaired t-tests. ns, *p* > 0.05; *, *p* < 0.05; ***, *p* < 0.001.

The results show that 2 days after TLR3 activation in the striatum, there were significant increases in the levels of full-length and cleaved complement C3 protein (Fig. 1A-C). TLR3 activation increased the ratio of cleaved complement C3 fragments to uncleaved protein (Fig. 1D), indicating that there is increased activation of complement C3 protein in response to TLR3 activation. Corresponding changes in levels of the upstream complement factor C1q were not observed (Fig. 1E,G). Levels of monomeric α-synuclein protein in the striatum increased by 41% by 2 d after Poly(I:C) (Fig. 1E-F, *t*_(17)_ = 4.710, *P* = 0.0002). The increased levels of complement C3 and α-synuclein were early acute events that occurred prior to detectable increases in astrocyte and microglial activation (Fig. 1E,H–I). These data indicate that the complement pathway is engaged in the early inflammatory response to a direct viral-like stimulus in the brain that is linked to increases in α-synuclein protein levels.

### C3 splice-switching ASO results in downregulation of *C3* transcript in the mouse striatum in vivo

Knockdown of *C3* expression is an effective way to block all arms of the complement system, namely, the inflammatory action of the anaphylatoxins C3a and C5a, and the terminal lytic pathway^51^. To assess the effect of complement pathway inhibition in the in vivo Poly(I:C) model, a splice-switching ASO specific for *C3* (C3 ASO) was utilized as a genetic tool to manipulate its expression. The aim of the experiment was to block C3 activation prior to TLR3 activation to investigate how reduced complement activation may prevent downstream inflammatory and cellular reactions. Given that at baseline, complement C3 is not active in the brain tissue, mice in the absence of an inflammatory trigger by Poly(I:C) were not studied with C3 inhibition. The C3 ASO was designed to be complementary to 5’ splice site of exon 2 of the *C3* pre-mRNA, to block splicing at the site. C3 ASO blocks splicing at the targeted site, which activated splicing of an upstream 5’ splice site in exon 2. Activation of this cryptic splice site resulted in a shift in the open reading frame and the introduction of a premature termination codon (PTC) in exon 3. This frame-shifted *C3* mRNA is expected to be degraded by the cellular nonsense-mediated mRNA decay (NMD) system, thus effectively reducing gene expression. A non-target ASO (NT ASO) that is not complementary to any RNA transcript of the mouse genome was used as a negative control.

**Figure 2:**
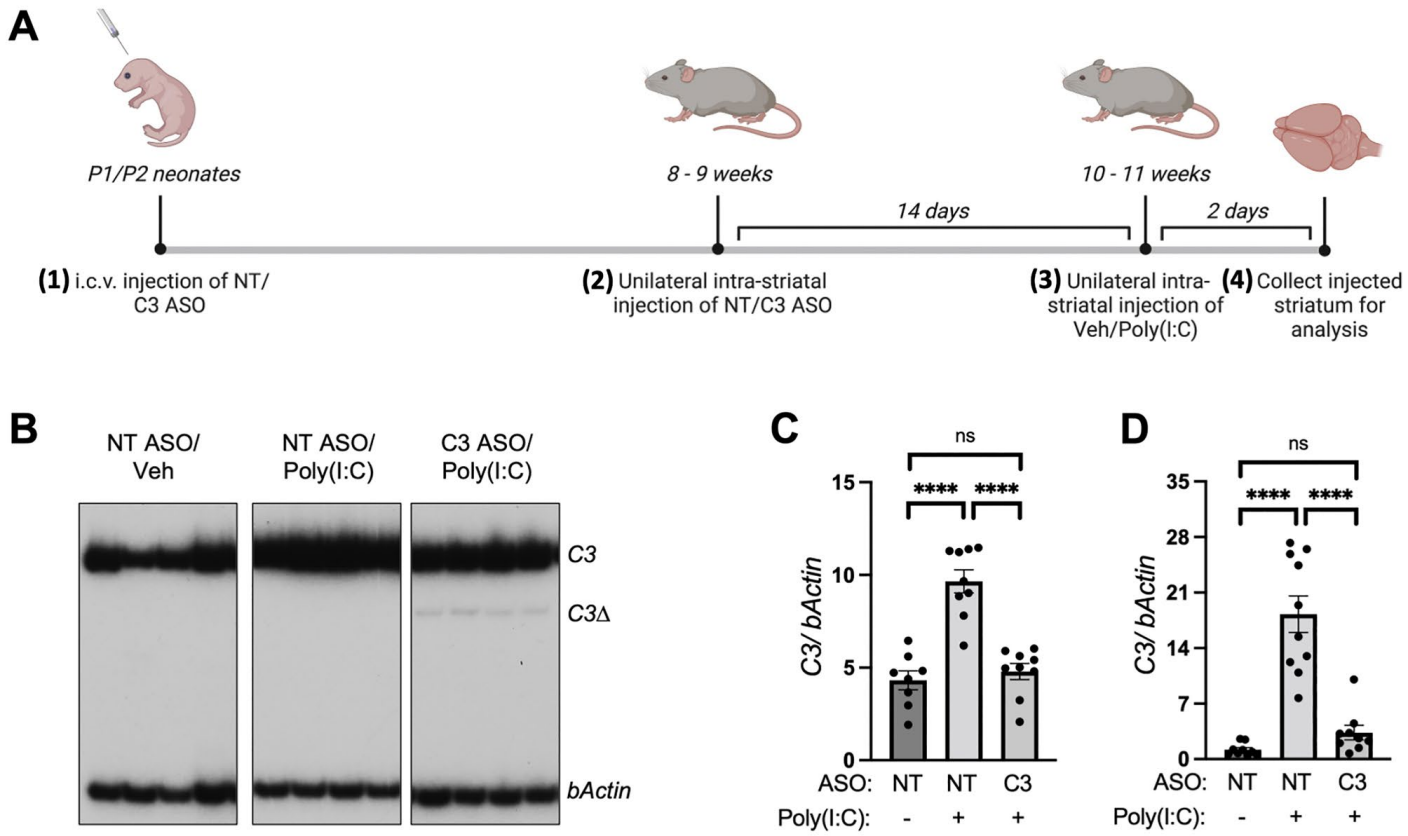
Splice-switching C3-specific ASO exhibits target engagement and downregulation of *C3* transcript in vivo. (**A**) Overview of experimental paradigm for C3-ASO and dsRNA delivery into mice: (1) Neonatal mice at postnatal day 1 or 2 (P1 or P2) were injected with a first dose of (50 μg/animal) non-target (NT) or complement C3 specific ASO into the cerebral lateral ventricles (i.c.v. injection). (2) After 8–9 weeks, a second dose of NT or C3 ASO was administered unilaterally to the right striatum of the adult mice (3 deposits per striatum, 40 μg/deposit). (3) 14 days after administration of the second dose of ASO, the mice were injected with Vehicle (Veh) or Poly(I:C) (50 μg) directly into the striatum. (4) Mice were euthanized 2 days post Poly(I:C) administration, and injected striatum was collected for further RNA and protein analyses between NT ASO/Veh (n = 9), NT ASO/Poly(I:C) (n = 10) and C3 ASO/Poly(I:C) (n = 10) groups. (**B**, **C**) RT-PCR and (**D**) qPCR measurement of *C3* RNA from striatum tissues of mice injected with NT or C3 ASO and co-administered with Veh or Poly(I:C). Representative, high exposure gel images are shown in (**B**) to visualize truncated *C3* transcript (*C3Δ*). The uncropped gel can be found in Fig. S8. Quantitation in (**C**) was performed using low exposure images. Data represented as arithmetic mean ± SEM. Statistical analysis was performed using one-way ANOVA followed by Tukey’s post hoc analysis. ns, *p* > 0.05; ****, *p* < 0.0001.

RT-PCR performed on the RNA isolated from striatum tissues showed that in response to intra striatal Poly(I:C) administration, a 2.23-fold significant elevation in total C3 mRNA transcript was observed in mice co-administered with NT ASO, while no change in *C3* transcript was observed in mice co-administered with the C3 ASO (Fig. 2B–C, F_(2,23)_ = 31.25, *P* < 0.0001). The data was further confirmed using an independent RT-qPCR experimental analysis (Fig. 2D, F_(2,25)_ = 38.04, *P* < 0.0001). Administration of the splice-switching C3 ASO led to the production of a spliced isoform of *C3* mRNA (*C3Δ*) in all the C3 ASO injected animals, and a reduction of the total *C3* mRNA transcript levels (Fig. 2B). *C3Δ* isoform constituted a small percentage of the total *C3* transcript, and this is likely due to this specific isoform being degraded by NMD. Taken together, these data show that the C3-specific splice-switching ASO exhibits effective target engagement and lowers *C3* transcript expression in the CNS in vivo.

### Reduction of TLR3 induced cytokine and chemokine levels by the inhibition of complement C3 in the mouse striatum

**Figure 3:**
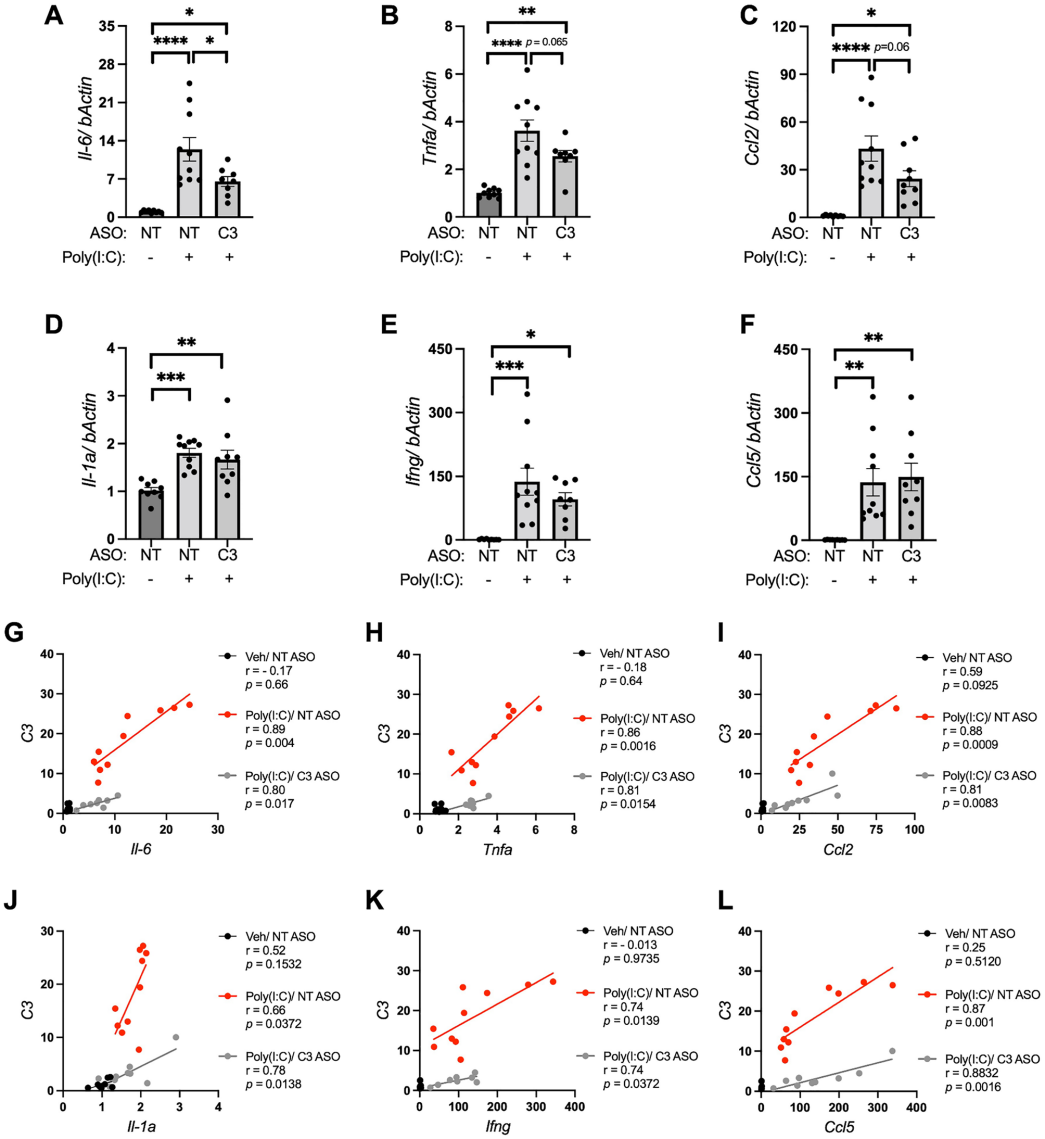
Knockdown of *C3* reduced cytokine and chemokine responses induced by TLR3 activation. qPCR analysis to measure transcript levels of (**A**) *Il-6* (**B**) *Tnfa* (**C**) *Ccl2* (**D**) *Il-1a* (**E**) *Ifng* and (**F**) *Ccl5* in the striatum of mice from NT ASO/Veh (n = 9), NT ASO/Poly(I:C) (n = 10) and C3 ASO/Poly(I:C) (n = 10) experimental groups. Correlation analyses between *C3* transcript levels and (**G**) *Il-6* (**H**) *Tnfa* (**I**) *Ccl2* (**J**) *Il-1a* (**K**) *Ifng* and (**L**) *Ccl5* transcript levels were performed and Pearson’s correlation coefficients are shown. Error bars represent SEM. Statistical analysis of data from figures (**A–F**) was performed using one-way ANOVA followed by Tukey’s post hoc analysis. *, *p* < 0.05; **, *p* < 0.01; ***, *p* < 0.001; ****, *p* < 0.0001.

Induction of inflammation, chemotaxis, and cytokine production by the action of the anaphylatoxins C3a and C5a constitute immediate downstream effects of complement pathway activation^52^. To examine the effect of *C3* knockdown on cytokine and chemokine levels, a transcript analysis was performed using RT-qPCR on RNA isolated from striatal tissues. Transcript levels of cytokine and chemokines were used in this study as a proxy for protein analysis given the small brain tissue regions analyzed. The data showed that mRNA levels of all the assessed cytokines and chemokines, *Il-6* (12.32-fold), *Tnfa* (3.58-fold), *Ifng* (10.53-fold), *Il-1a* (1.78-fold), *Ccl2* (40.17-fold) and *Ccl5* (126.24-fold) were significantly elevated in Poly(I:C) injected mice compared to vehicle controls (Fig. 3A–F). Interestingly, mice with reduced expression of *C3* (C3 ASO injected mice) exhibited significantly reduced level of *Il-6* elevation and a strong trend for reduced expression of *Tnfa* and *Ccl2* in response to Poly(I:C) compared to animals with wild type level of *C3* (NT ASO injected mice) (Fig. 3A–C). No such altered response to Poly(I:C) was observed with *Ifng*, *Il-1a* and *Ccl5*. To further assess the association between *striatal C3* transcript levels with levels of each of the cytokines and chemokines within each group of animals (Veh/NT ASO, Poly(I:C)/NT ASO, Poly(I:C)/C3 ASO), correlation analysis was performed between the variables. Cross correlation analysis revealed a significant positive correlation between *C3* transcript and *Il-6*, *Tnfa*, *Ccl2*, *IL-1a*, *Ifng* and *Ccl5* in both Poly(I:C)/NT ASO and Poly(I:C)/C3 ASO groups, showing that elevation in *C3* level is associated with elevation in these cytokines and chemokines within the brain of these animals (Fig. 3G–L).

### Antisense-mediated reduction of C3 prior to TLR3 activation blocked increases in α-synuclein protein levels in the mouse striatum

**Figure 4:**
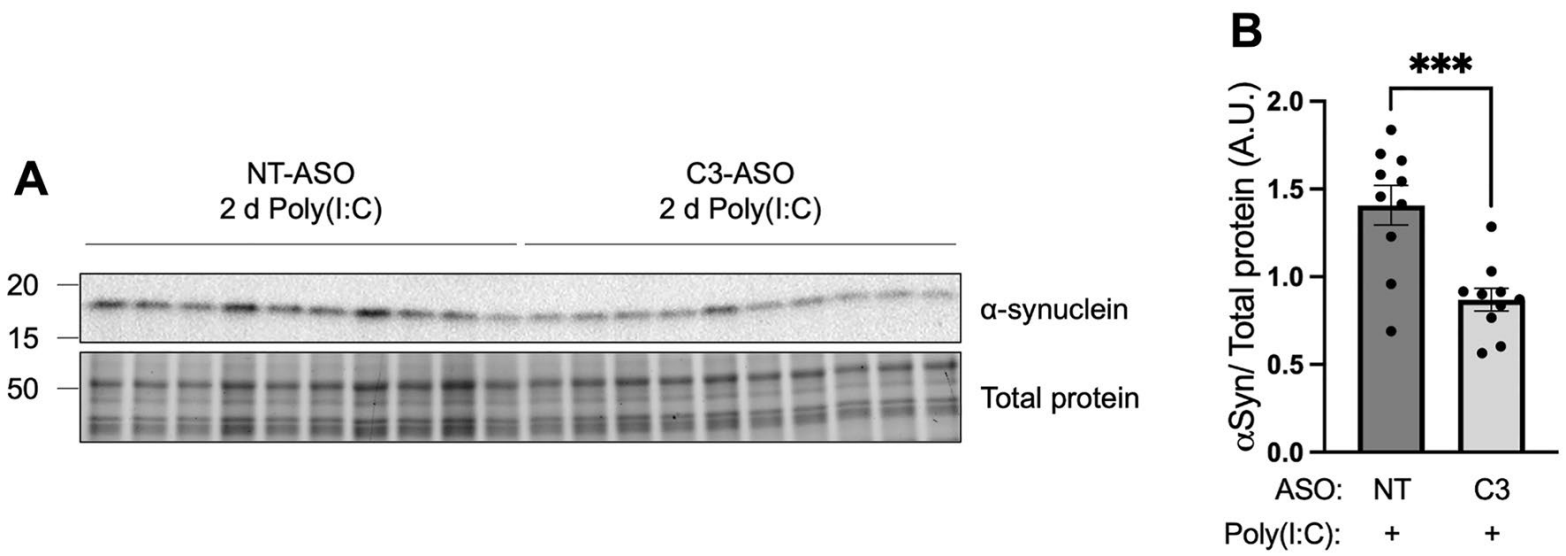
Knockdown of C3 prior to TLR3 activation reduced α-synuclein protein levels in the striatum of mouse brain. Alpha-synuclein levels in the injected striatum of mice from NT ASO/Poly(I:C) (n = 10) and C3 ASO/Poly(I:C) (n = 10) groups were measured by Western blot (**A**). The uncropped blot can be found in Fig. S9. (**B**) Histogram bars represent the mean levels of α-synuclein normalized to total protein loading. Error bars represent SEM. A.U., arbitrary units. Statistical analysis was performed using unpaired t-tests. ***, *p* < 0.001.

TLR3 activation caused early acute activation of complement C3 with corresponding elevations of inflammatory cytokines and α-synuclein levels. To determine if reducing brain inflammation by knockdown of complement *C3* could reduce α-synuclein levels that increase following TLR3 activation, striatum α-synuclein levels were quantified from mice that received C3 ASO prior to Poly(I:C) compared to mice that received NT-ASO. Monomeric α-synuclein levels were reduced when complement *C3* was reduced prior to TLR3 activation, relative to control mice that received NT ASO (Fig. 4A,B, *t*_(18)_ = 4.154, *P* = 0.0006).

## Discussion

### Summary of experiments and findings

In this in vivo brain model of neuroinflammation, direct activation of brain TLR3 using the synthetic viral dsRNA mimetic, Poly(I:C), caused acute activation of complement C3 and increased α-synuclein protein levels. Complement *C3* activation correlated with the production of potent inflammatory factors, including cytokines *Il-6*, *Tnfa*, *Il-1a* and *Ifng*, and chemokines *Ccl2* and *Ccl5*. The aim of the study was to understand how the inflammatory and cellular responses downstream of TLR3 may be prevented by blocking the activation of complement C3. The results showed that increased levels of inflammatory cytokines and α-synuclein protein in response to TLR3 activation were prevented by an ASO that blocked the production and activation of complement *C3*. This is the first demonstration to our knowledge that α-synuclein increases are downstream of complement C3 activation and occur downstream of TLR3 activation*.* These data point to important and critical interactions between brain inflammatory cytokine and complement systems and α-synuclein changes. Such interactions are also seen in viral encephalitis and with other inflammatory triggers, which can precipitate pathological changes associated with PD and LBD^1, 2^.

### Viral-like TLR3 activation increases downstream complement and inflammatory cytokine responses

Our study shows that brain TLR3 activation by Poly(I:C) significantly increased full length as well as cleaved complement C3 levels suggestive of complement pathway activation. Cleavage of C3 is an essential step in the complement activation cascade. Deposition of complement C3 is reported in the human brain after viral infection, including SARS-CoV-2 and HIV^15,60^. The complement cascade can be initiated by the C1q-dependent classical pathway or the C1q-independent alternative and lectin pathway^52^. Interestingly, administration of Poly(I:C) did not alter the level of C1q in our study, suggesting that complement pathway activation in this model is likely to occur through one of the C1q-independent activation pathways. Supporting this hypothesis, previous studies have shown that treatment of in vitro macrophage cells and colonic epithelial cells with Poly(I:C) significantly upregulates complement factor B (CFB), an upstream component of the alternative pathway that forms a C3 convertase enzyme leading to cleavage of full length C3^61,62^. Furthermore, intra-tracheal administration of Poly(I:C) in mice led to robust upregulation of *CFB* transcript in alveolar macrophages 6 h post injection, showing that alteration in CFB levels, and consequent activation of the alternative pathway constitute an acute response to Poly(I:C)^61^. Consistent and complementary to this data, CFB levels were also elevated in patients with inflammatory bowel disease, where TLR3 has been linked to have a pathological role^62^. Taken together, these findings suggests that activation of the complement pathway in response to Poly(I:C) in mouse brain that we observe in our study could occur through the activation of the alternative complement pathway.

In addition to elevation of complement C3, in our study, intra-striatal TLR3 activation also led to significant elevations in transcript levels of the proinflammatory cytokines *Il-6*, *Tnfα*, *Ifng* and *Il-1a*, and the chemokines *Ccl2* and *Ccl5*, demonstrating that TLR3 activation leads to robust inflammatory responses in the brain. Blockade of C3 significantly reduced levels of *Il-6* in response to Poly(I:C), suggesting the regulation of Il-6 by C3. Importantly, complement *C3* levels showed a significant positive correlation with all measured cytokines and chemokines in Poly(I:C) injected animals. Consistent with this data, a previous study showed that intra-nigral administration of Poly(I:C) in rats led to local elevations of IL-1α, CCL2 and CCL5 in the SN and TNFα and CCL2 in the striatum which peaked at 12-days post injection^5^. These cytokine peaks were observed in the context of TLR3 protein upregulation, and nigral and striatal microglial and astrocyte activations beginning at 4 days, which peaked after 12 days and persisted for 33 days^5^. In that study, assessment of microglia phenotypic markers demonstrated Iba1-labeled microglia with short and thick processes and large cell bodies, that also expressed markers for major histocompatibility complex-II (MHC-II), demonstrating microglial activation^5^. The new data in the current study, showing a robust acute induction in cytokines and chemokines as early as 2-day post injection of the dsRNA mimetic, Poly(I:C), prior to biochemically detectable microglial and astrocyte activations, support the idea that both early and prolonged cytokine and chemokine responses in the brain are potentially involved in long-term brain changes. A marked increase in activated microglia (MHC-II-positive) in the brain are also reported in patients with PD^63^.

Coronaviruses, including SARS-CoV-2, produce dsRNA early during the infection cycle, which is recognized by the viral dsRNA receptor, TLR3. In the current study, the synthetic dsRNA, Poly(I:C), was used to experimentally model innate brain immune responses to viral-like infection in the brain. Elevation of pro-inflammatory cytokines and complement activation in the brain as demonstrated in the current study following delivery of the dsRNA viral mimetic have also been reported in the brain and CSF of patients with COVID-19^12–15^. SARS-CoV-2 infection causing COVID-19 can lead to long-term neurological and psychiatric manifestations^21,22^. As previously hypothesized^1,64^, a recent analysis of two biomedical databases showed that flu infections and flu-induced pneumonia are associated with a two- to sevenfold- increased risk for neurodegenerative disease, including PD, AD, dementia and ALS^11^. Further studies to examine the effects of prolonged or repeated challenges of dsRNA in experimental in vivo systems over time are warranted in order to understand long-term cellular adaptations and responses in the brain that may lead to subsequent neurological and psychiatric deficits following viral infections.

Activation of TLR3 signaling leads to activation of the nuclear factor κb (NF-κb) and IFN regulatory factor 3 (IRF3) signaling pathways eventually leading to the production of proinflammatory cytokines and chemokines^65^. The potent anaphylatoxins C3a and C5a produced by the complement pathway activation also mounts immune response by the secretion of cytokines. To delineate the cytokine and chemokine responses induced by the complement pathway and by TLR3 activation in this Poly(I:C) model, we utilized genetic inhibition of C3 using C3 specific ASOs. Our data shows that *Il-6*, a proinflammatory cytokine, which was elevated upon TLR3 activation by Poly(I:C) was significantly reduced in animals with lower *C3* levels. A strong trend for a similar relationship was also observed with the cytokine, *Tnfa*, and the chemokine, *Ccl2*, indicating their potential regulation by the complement pathway. However, the treatment-induced reduction of *C3* did not change the levels of *Ifng*, *Il1a* and *Ccl5* in the striatum, indicating that these cytokines are not directly regulated by C3 and are independently initiated by other factors downstream of TLR3. Taken together, these findings clearly demonstrate that the production and activation of complement C3 occurs downstream of TLR3 activation in vivo, and that activated complement C3 is responsible for a proportion of the inflammatory response, that includes *Il-6*, to viral-like stimuli.

### The potential role for α-synuclein in brain TLR3-mediated inflammation

Α-synuclein is a lipid membrane-binding protein that is enriched in synaptic terminals of neurons and has important physiological functions related to synaptic function, including synaptic vesicle binding and trafficking, neurotransmitter release, synaptic activity, and plasticity^28,66^. The accumulation and aggregation of α-synuclein in neurons is associated with neuropathology in PD and LBD and can be induced by inflammatory triggers^30,36, 67,68^. The current data showed that viral-like TLR3 activation in the brain, simulating inflammatory complement, cytokine and chemokine elevations, induced an acute elevation of α-synuclein protein levels. These early increases in monomeric α-synuclein pools in the context of complement and cytokine activations after TLR3 activation, which is known to lead to subsequent peaks in glial activation and inflammation during a cytokine storm^5^, potentially increase the risk of long-term α-synuclein neuropathology associated with damage and loss of vulnerable neurons. Elevated α-synuclein expression in the brain and gastrointestinal neurons has been reported following viral infections in rodent models as well as in humans^30,33,35,36,69^. Recent reports demonstrate that α-synuclein is also increased in the brain in rodent and non-human primate experimental models of SARS-CoV-2 infection^38,39^. Knockdown or deficiency of α-synuclein in rodents induces nigral dopaminergic neuron loss degeneration^70^ and increased viral load in the brain following viral infection^67^. Such acute inflammatory activations and elevated α-synuclein are likely efficiently handled and resolved by the brain, however, sustained or repeated inflammatory stimuli, combined with environmental and genetic factors, could prevent the resolution of inflammatory and protein responses in the brain^71^, and eventually lead to reduced clearance of α-synuclein, as well as other aggregation-prone proteins associated with neurodegenerative disease pathology^27,28^. Patients with post-COVID-19 syndromes exhibit elevated neurofilament-light and phosphorylated Tau levels, and impaired amyloid processing in the CSF, further illustrating an association between viral-inflammatory responses and increases in proteins that are linked with neurodegenerative disease^72,73^.

In addition to neurotropic viruses that directly infect neurons in the CNS that can cause severe brain inflammation, more common peripheral viral infections, such as by respiratory viruses, can enter the brain via infection of the peripheral nervous system or by infiltration through the BBB. Such peripheral viral infections can cause systemic cytokine storm that is accompanied by central cytokine elevations^1,74^. However, a likely scenario that is supported by recent data from COVID infections indicate that generalized viral infections can also trigger inflammatory cascades indirectly in the brain. In such cases, repeated viral infections, even flu viral syndromes would generate peak levels of cytokines that would engage a microglial and immune response also in brain tissue^1,2,64,75^. Under such circumstances, sustained, or severe central or peripheral infections that cause prolonged activation of inflammatory factors in the brain could cause long-term elevations in α-synuclein levels that promote its aggregation into unresolvable inclusions^27,28^.

Notably, the increase in α-synuclein in the striatal brain region observed following TLR3 activation was prevented by prior knockdown of complement *C3*. This is further evidence that α-synuclein cellular functions are induced by inflammation^30,33–36,69,76^. Interestingly, it was previously shown that sustained lipid elevations in the substantia nigra of mice causes α-synuclein aggregation^6^. Such glycolipid changes induced elevated α-synuclein levels, which were associated with complement and cytokine-mediated inflammation. In this context, it is important to note that bacterial-like TLR2 and TLR4 activations can also increase α-synuclein levels^30,77,78^. In summary, there are multiple lines of evidence showing that α-synuclein expression can be induced by inflammatory activation, and the data in the current study further demonstrates that α-synuclein increases are an acute response to viral-like infection and are downstream of complement C3 activation.

### Relevance of the observed complement and cytokine elevations to the pathophysiology of neurodegenerative diseases

The experiments reported here show that elevation of complement C3 constitutes an acute response to TLR3 activation. In addition to its role in inflammation, elevation of C3 has been implicated in multiple models of neurodegeneration and its inhibition was shown to be protective against neurite loss^79–82^. Significant elevation in C3 levels were observed in the post synaptic density fraction isolated from superior frontal gyrus, and in the CSF of AD patient samples compared to healthy subject controls^79^. In the same report, elevated C3 levels were also observed in hippocampal glial cells in rodent models of tauopathy (TauP301S) and amyloidosis (PS2APP). Genetic deletion of C3 led to amelioration of plaque-associated spine loss in PS2APP mice, and brain atrophy and hippocampal neuronal changes in TauP301S mice^79^. Previous studies in early-stage experimental autoimmune encephalomyelitis (EAE) model of multiple sclerosis (MS) identified that complement C3 was significantly elevated in the hippocampus^83^ and activated microglia in the dentate gyrus (DG)^80^. Experiments involving pharmacological inhibition of C3 by Rosmarinic acid and genetic inhibition using C3 deficient (C3 KO) mice was adequate to rescue the reduction in dendritic length and density in the DG granule neurons, and memory impairments observed in this model, thus suggesting a role for microglial C3 in hippocampal neurodegeneration^80^. An interesting role for C3 in aging was identified when C3 deficient C57BL/6 mice were found to be protected against age-related, region-specific, synapse and neuronal loss and cognitive decline^81^. Taken together, these experiments provide compelling data for considering the action of complement C3 in neuronal and brain degenerative disease processes. While our study showed elevation in striatal C3 level as an acute response to the inflammatory TLR3 agonist Poly(I:C) in wild type mice, we believe that repeated or prolonged inflammatory changes due to genetic and environmental factors can be detrimental to neuronal cells^2,27,84^.

The induction of complement activation and subsequent cytokine changes linked to acute inflammatory responses can increase the vulnerability of many neuronal systems in certain brain regions^1,27^. Previous studies have shown that increased cytokine levels can make the midbrain dopaminergic system vulnerable to oxidative stress, suggesting that inflammatory pre-conditions can accelerate degeneration associated with PD and LBD^4,5^. Conversely, blockade of inflammatory cytokines can prevent such dopamine neuron degeneration^4,5,29^. Supporting a causality of increased inflammatory and cytokine activation on PD incidence, are epidemiological studies demonstrating that chronic use of anti-inflammatory drugs such as NSAIDs and immunosuppressants lower the risk for developing sporadic PD in humans^85^, and showing an association between inflammatory bowel disease and increased incidence of PD^86,87^. Inflammatory cytokine network activations, such as those observed following viral-like TLR3 activation in the current study, have been reported in vulnerable brain regions, CSF and serum of patients with AD, PD and related dementia syndromes^88–94^. For example, region specific elevation of IL-6 was observed in the caudate and putamen of PD patients^89^ and in the Aβ plaques in the cingulate cortex of AD brains compared to healthy subject controls^94^. Elevation of IL-6 in the CSF and serum was also reported in PD^88,93,95,96^ and AD patients^88,97^. A recent analysis of two biomedical databases reported that viral exposures increased subsequent later life risk of neurodegenerative diseases^11^. Systemic and brain inflammation in patients with COVID-19 is associated with elevated levels of the same pro-inflammatory molecules that were increased following experimental dsRNA exposure in the current study, including IL-6, TNF-α, and complement C3^14,15,75^. A meta-analysis that quantitatively summarized peripheral inflammatory cytokine levels in PD from 25 separate biomarker studies (encompassing 1547 PD patients and 1107 healthy controls) identified IL-6 to be one of the cytokines that is significantly elevated in PD compared to healthy subject controls^98^. A recent study that performed statistical evaluation between markers of inflammation and PD using a two-sample Mendelian randomization design identified that IL-6 levels exhibit causal association with the onset of PD^99^. Altered expression of IL-6 in the periphery and CNS is associated with neurodegenerative disorders and indicates that immunological events, specifically mediated by IL-6, in the pathophysiology of these diseases. The fact that the prevention of complement activation in the current study also reduce downstream IL-6 activation, is therefore interesting from the perspective of multiple neurodegenerative diseases that have IL-6 elevations in common.

### Supplementary Information

Supplementary Figures.

## Data Availability

The data generated during the current study are available from the corresponding author(s) on reasonable request.

## Abbreviations

6-OHDA: 6-Hydroxydopamine
AD: Alzheimer’s disease
ALS: Amyotrophic lateral sclerosis
AP: Anteroposterior
ASO: Antisense oligonucleotide
BBB: Blood brain barrier
BCA: Bicinchoninic acid
DG: Dentate gyrus
dsRNA: Double-stranded RNA
DV: Dorsoventral
EAE: Experimental autoimmune encephalomyelitis
EBV: Epstein-Barr virus
ECL: Enhanced chemiluminescence
EDTA: Ethylenediamine tetraacetic acid
GWAS: Genome-wide association study
i.c.v: Intracerebroventricular
KO: Knockout
LBD: Lewy body dementia
LPS: Lipopolysaccharide
MAC: Membrane attack complex
ML: Mediolateral
MSA: Multiple system atrophy
NMD: Nonsense-mediated mRNA decay
NSAID: Non-steroidal anti-inflammatory drug
NT: Non-target
PD: Parkinson’s disease
Poly(I:C): Polyinosinic:polycytidylic acid
PTC: Premature termination codon
PVDF: Polyvinylidene fluoride
RIPA: Radioimmunoprecipitation assay
RT: Reverse transcriptase
SDS-PAGE: Sodium dodecyl sulfate polyacrylamide gel electrophoresis
SEM: Standard error of mean
SNpc: Substantia nigra pars compacta
TLR3: Toll like receptor 3
