# Supplementary material for: Viral-like TLR3 induction of cytokine networks and α-synuclein are reduced by complement C3 blockade in mouse brain
Source: Sci Rep. 2023 Sep 13;13:15164. doi: 10.1038/s41598-023-41240-z (PMC10499893; doi:10.1038/s41598-023-41240-z)
Supplement: Supplementary file 1 — Supplementary Figures. [file 41598_2023_41240_MOESM1_ESM.pdf]

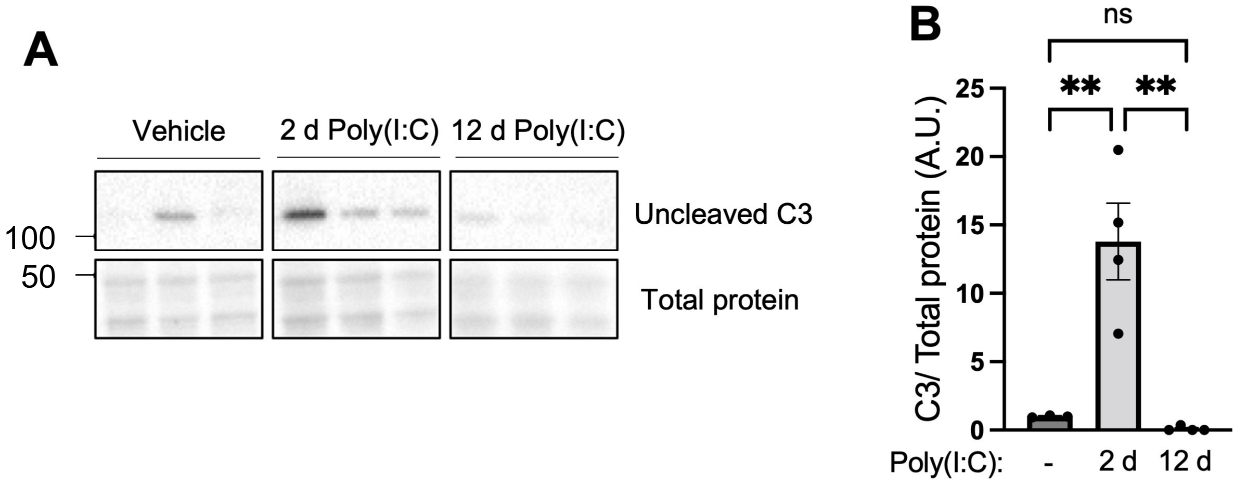

Sup. Figure 1. Intra-cranial TLR3 activation in mouse brain led to acute elevation of complement C3 in the striatum. WT mice received a unilateral deposit of 50 µg Poly(I:C) or vehicle in the striatum. Two and twelve days later, injected striata were isolated and homogenised in RIPA buffer for protein analysis. (**A**) A representative immunoblot image and (**B**) quantitation of complement C3 levels in striatal lysates 2 and 12 days post-Poly(I:C) injection are shown. Total protein level was used as a control to normalize for loading variability between samples. Error bars represent SEM, n=3-4 per group. Statistical analysis was performed using one-way ANOVA followed by Tukey's post hoc analysis. ns, $p>0.05$; **, $p<0.01$.

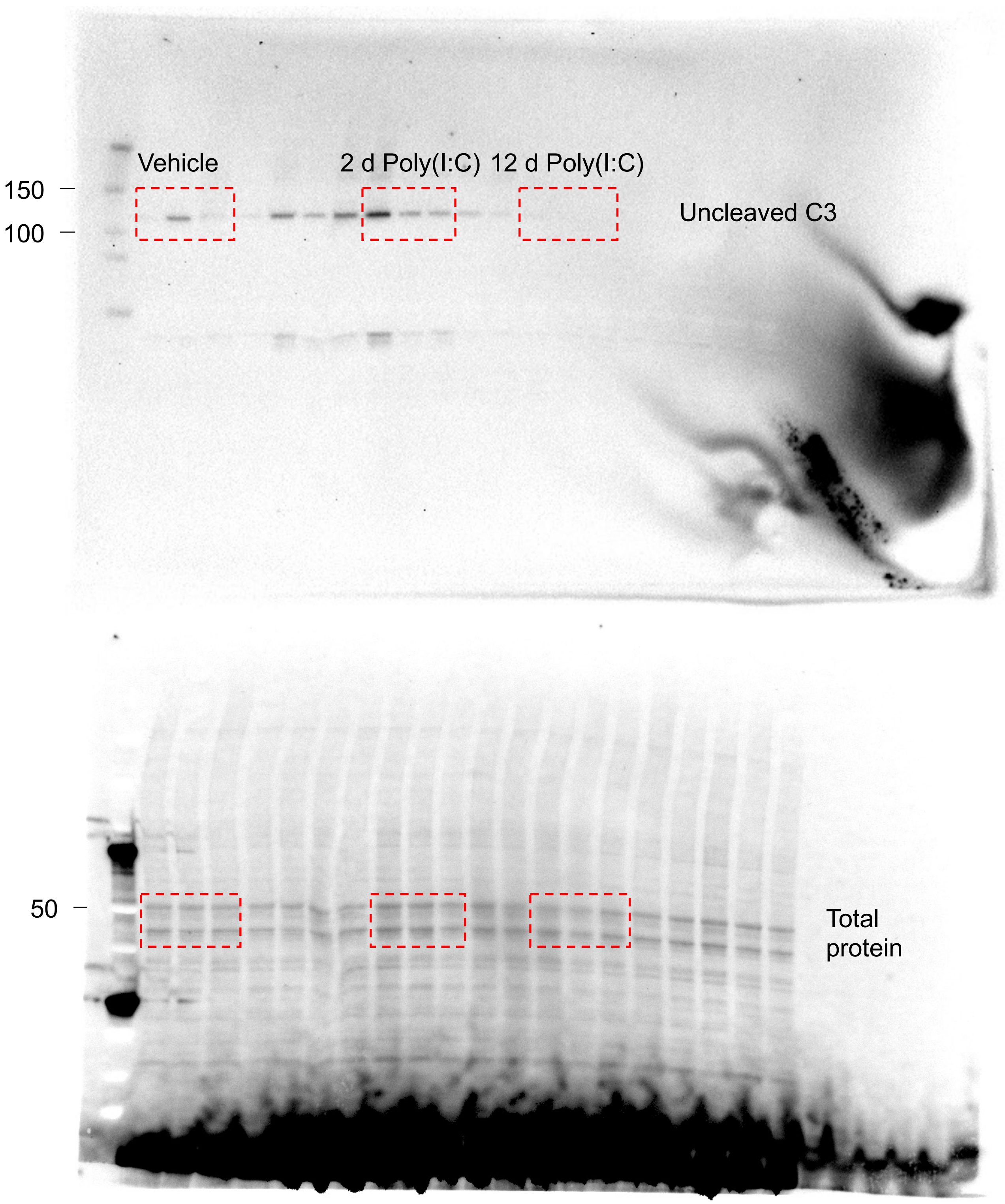

Sup. Figure 2. Intra-cranial TLR3 activation in mouse brain led to acute elevation of complement C3 in the striatum. Uncropped images of the complement C3 Western blot and protein loading are shown with red dotted boxes highlighting areas used to create the representative gel image in supplementary figure 1.

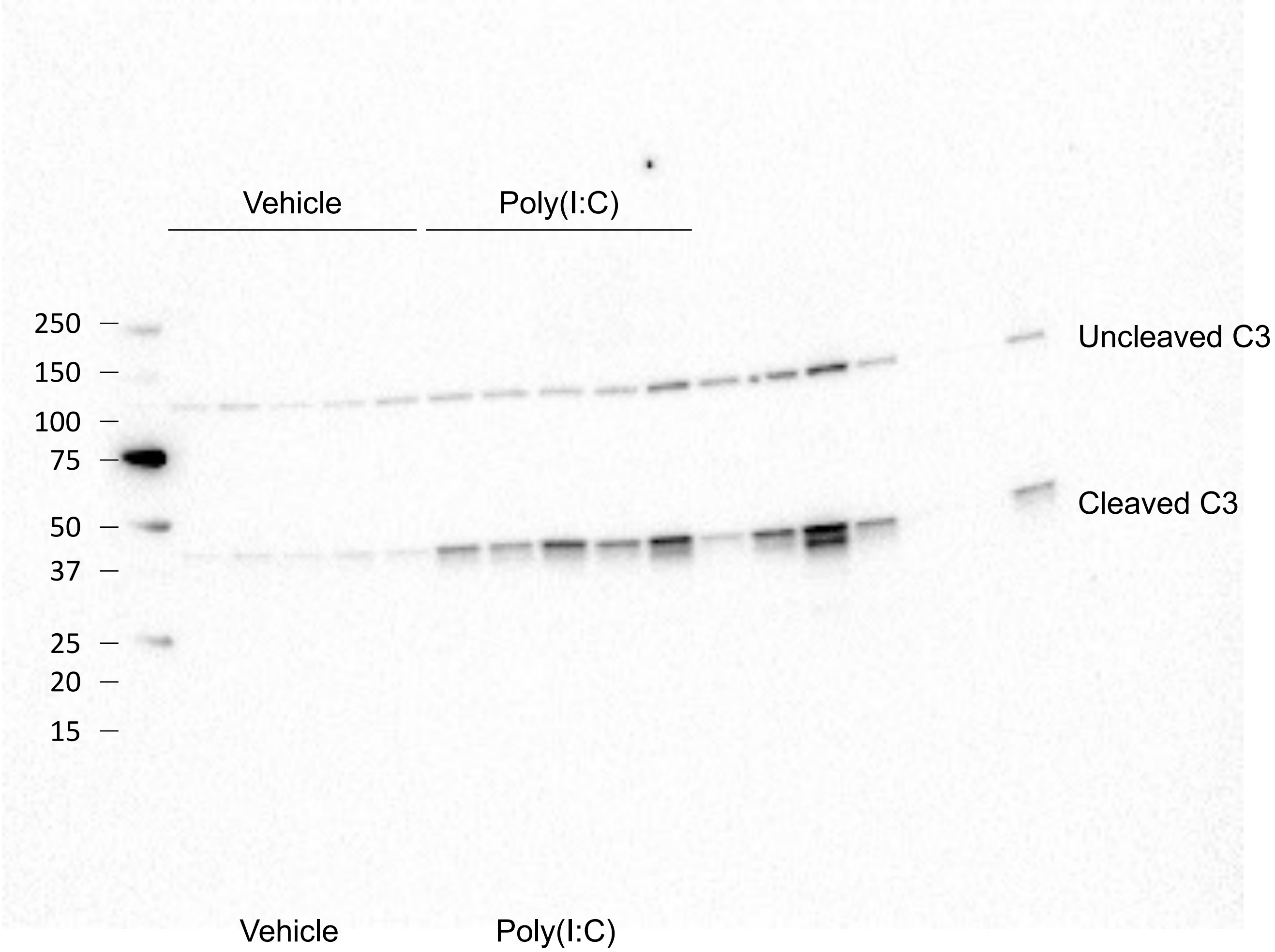

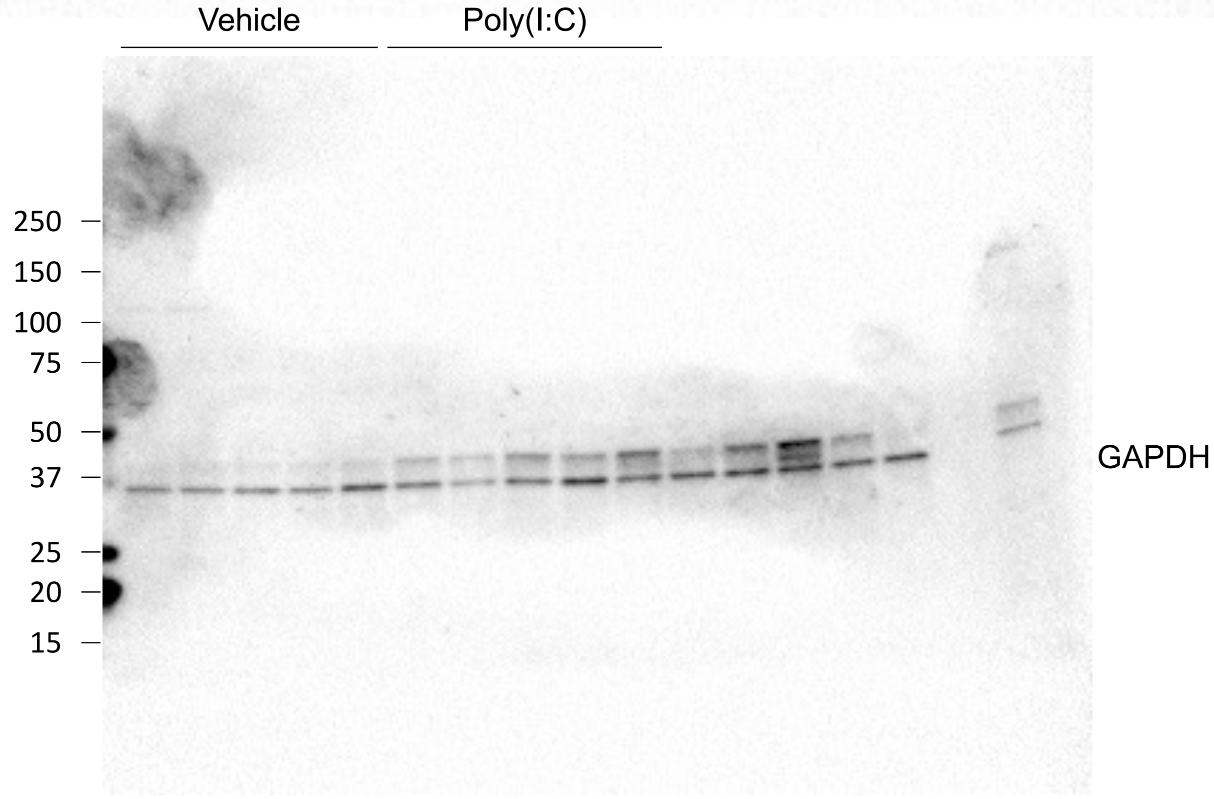

Sup. Figure 3. Intra-striatal TLR3 activation in mouse brain increased complement C3 protein levels. Uncropped images of the complement C3 and GAPDH Western blots used to create the representative gel image in figure 1A.

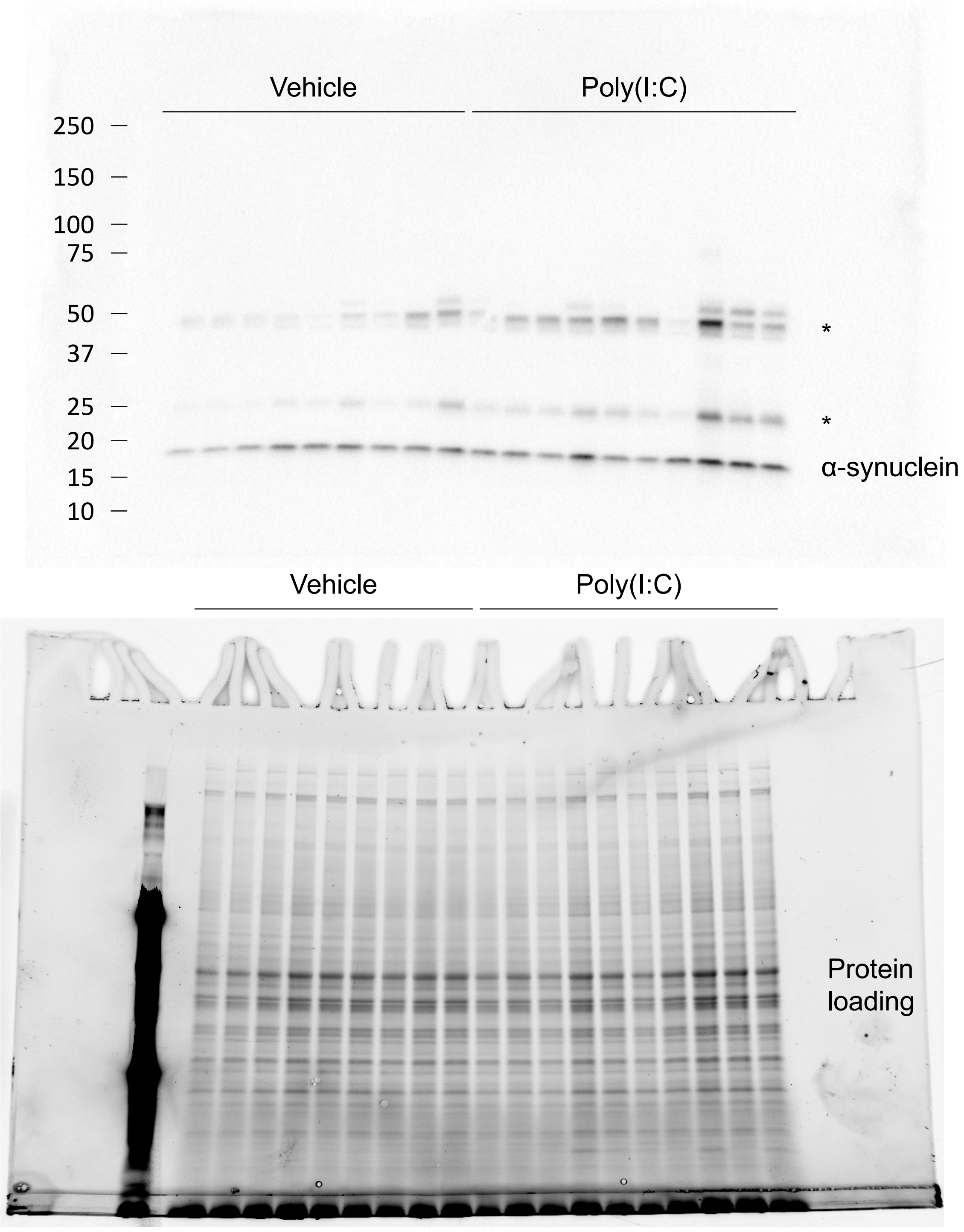

Sup. Figure 4. Intra-striatal TLR3 activation in mouse brain increased α-synuclein protein levels. Uncropped images of the α-synuclein Western blot and total protein loading used to create the representative gel image in figure 1E. Asterisks annotate non-specific bands that cross-reacted with secondary antibody.

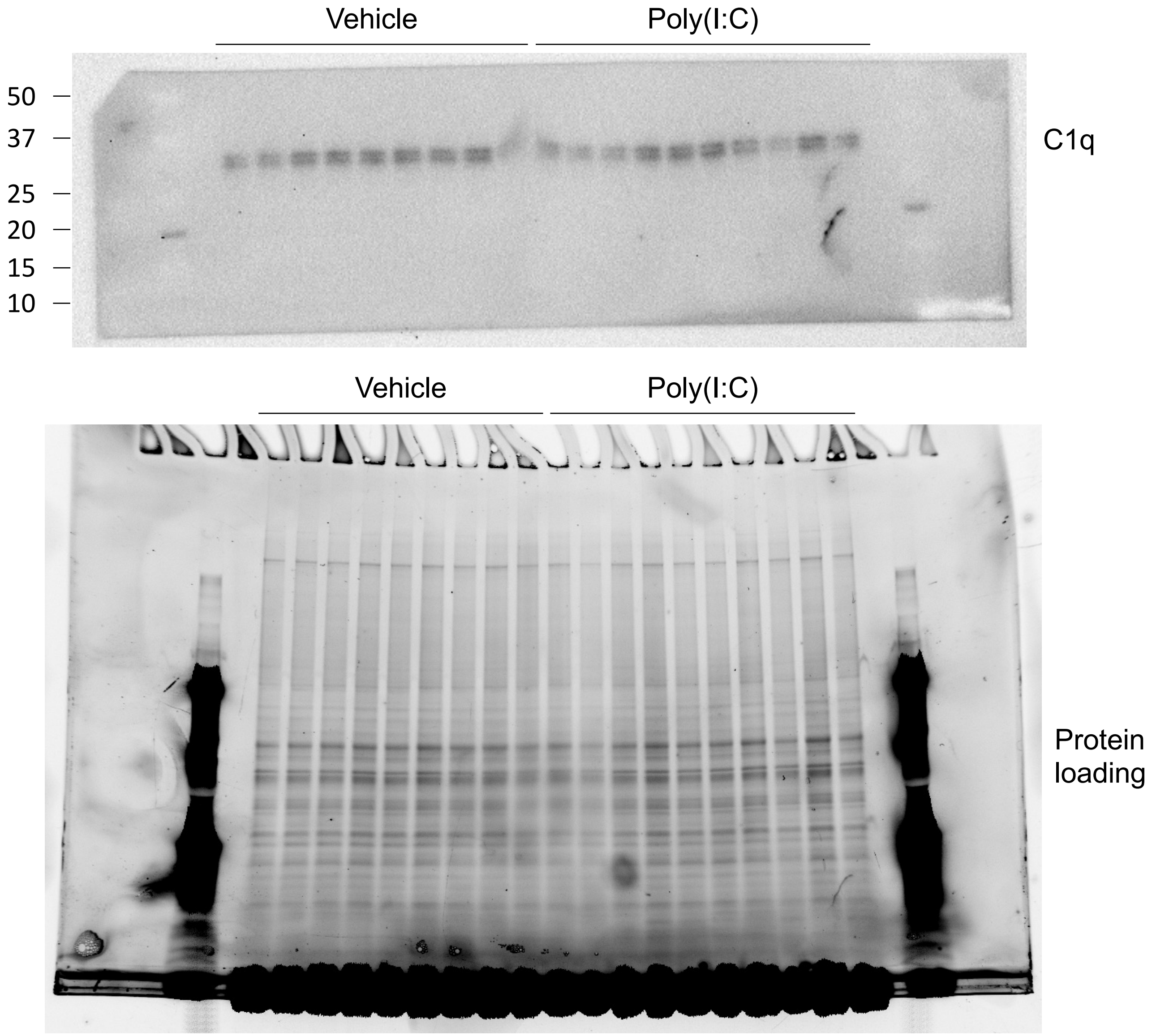

Sup. Figure 5. Intra-striatal TLR3 activation in mouse brain had no effect on C1q protein levels. Uncropped images of the C1q Western blot and total protein loading used to create the representative gel image in figure 1E.

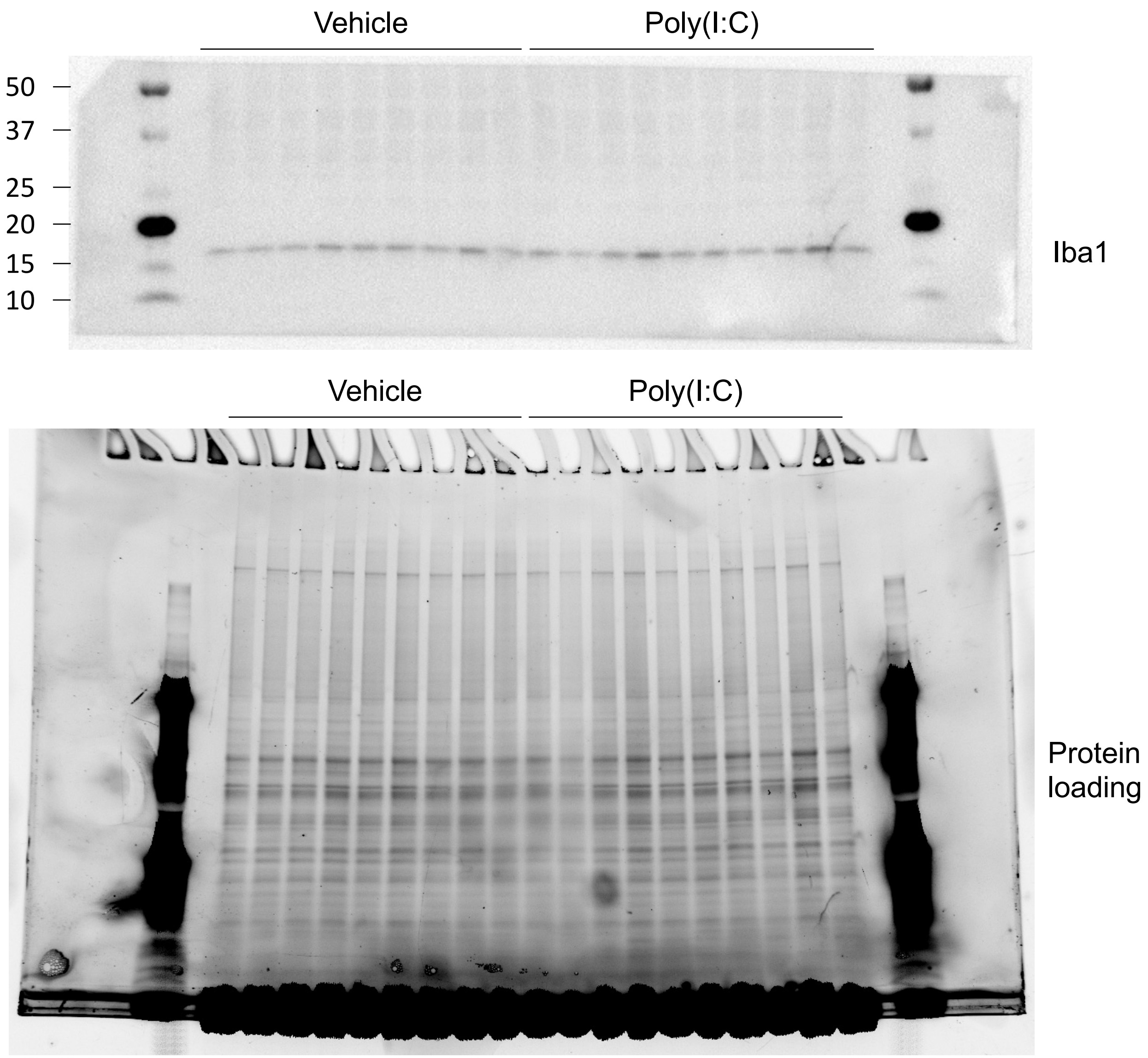

Sup. Figure 6. Intra-striatal TLR3 activation in mouse brain had no effect on Iba1 protein levels. Uncropped images of the Iba1 Western blot and total protein loading used to create the representative gel image in figure 1E.

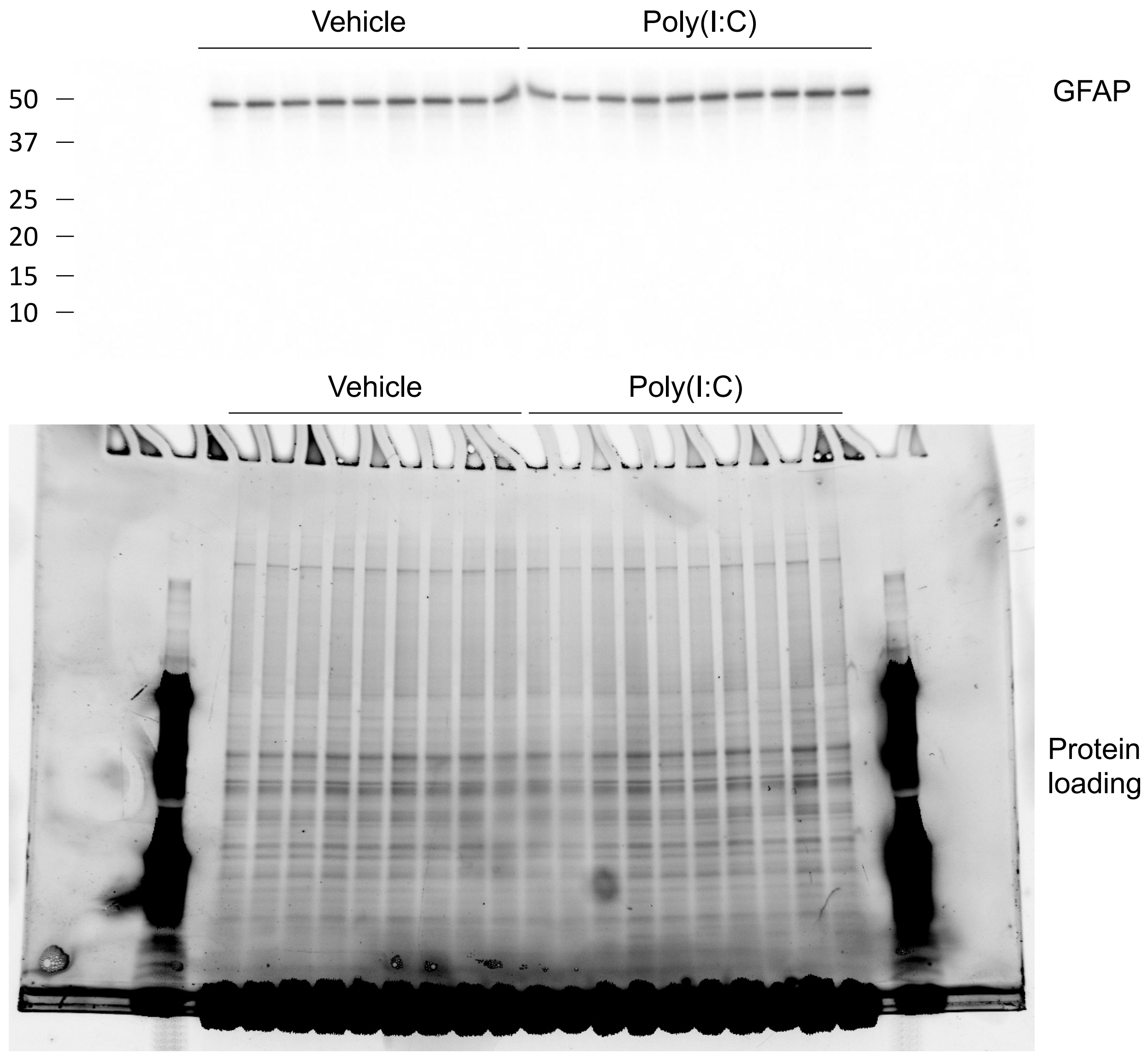

Sup. Figure 7. Intra-striatal TLR3 activation in mouse brain had no effect on GFAP protein levels. Uncropped images of the GFAP Western blot and total protein loading used to create the representative gel image in figure 1E.

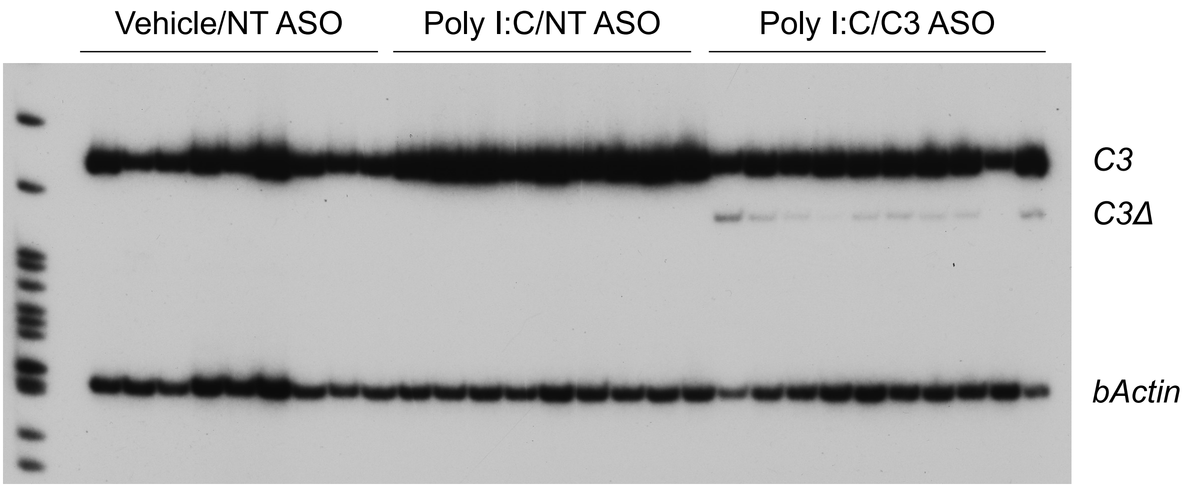

Sup. Figure 8. Splice switching C3-specific ASO exhibit target engagement and downregulation of *C3* transcript *in vivo*. Uncropped images of the complement C3 and bActin RT-PCR gel used to create the representative gel image in figure 2B.

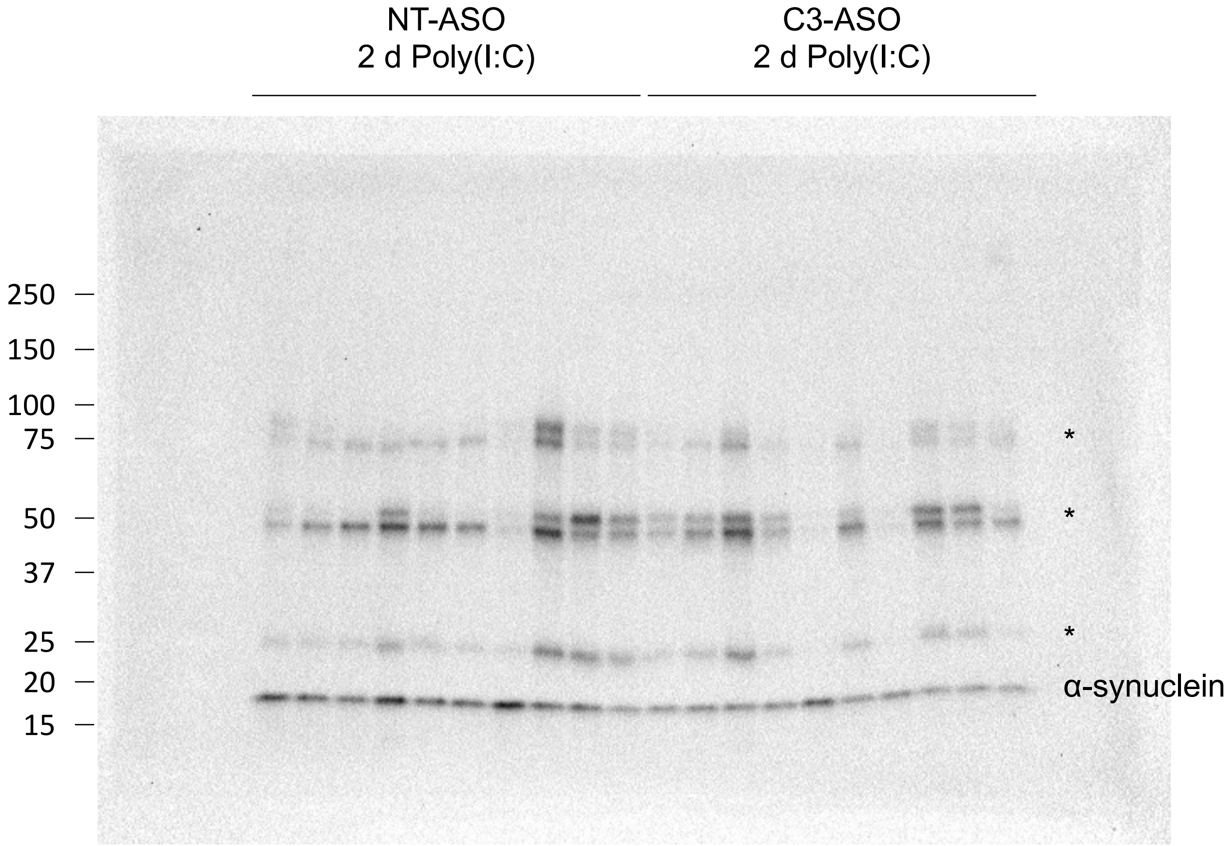

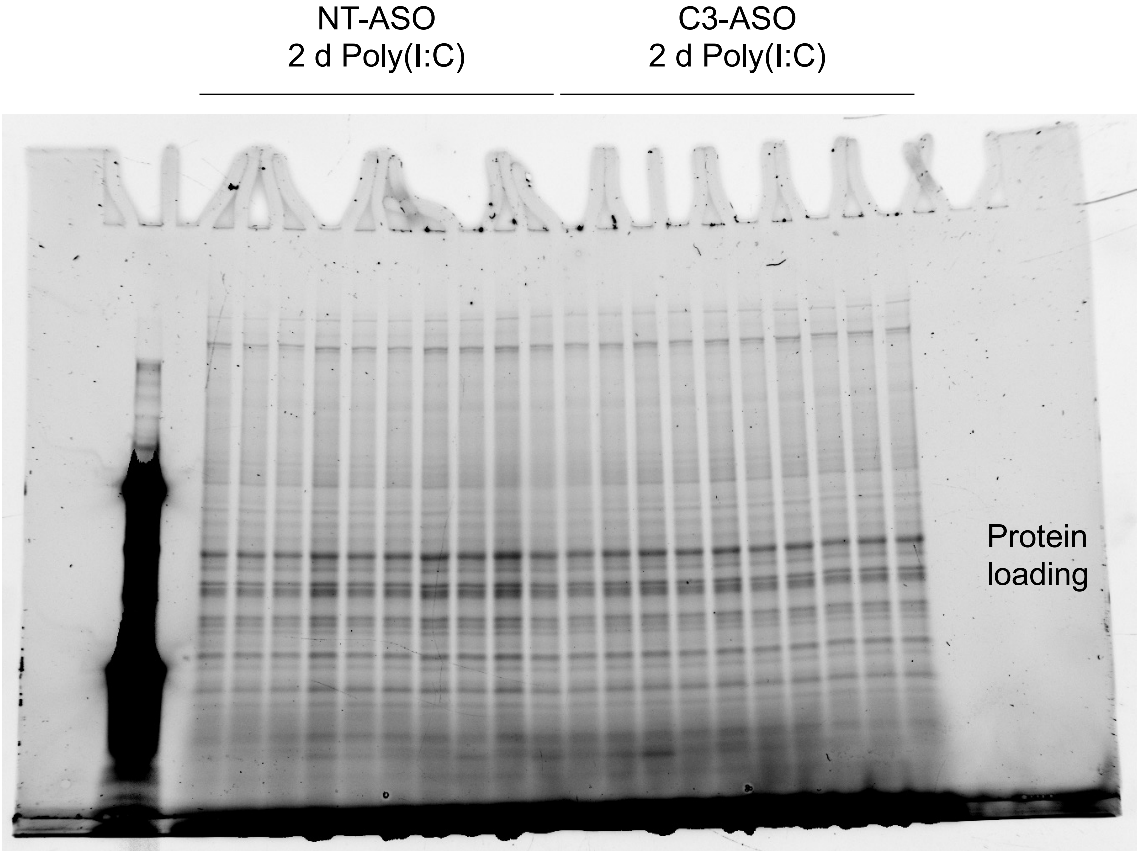

Sup. Figure 9. Knockdown of C3 prior to TLR3 activation reduced α-synuclein protein levels in the striatum of mouse brain . Uncropped images of the α-synuclein Western blot and total protein loading used to create the representative gel image in figure 4A. Asterisks annotate non-specific bands that cross-reacted with secondary antibody.
